# Advances in measurable residual disease assessment for acute myeloid leukemia: from cytogenetics and molecular biology to assessment of the methylation pattern and surface-enhanced Raman scattering as emerging technologies

**DOI:** 10.1186/s40364-025-00869-w

**Published:** 2025-11-28

**Authors:** Anamaria Bancos, Andrei Ivancuta, Vlad Moisoiu, Adrian-Bogdan Tigu, Diana Gulei, Madalina Nistor, Cristian-Silviu Moldovan, David Kegyes, Diana Cenariu, Mihnea Zdrenghea, Anca Bojan, Stefania D. Iancu, Nicolae Leopold, Gabriel Ghiaur, Horia Bumbea, Alina Tanase, Hermann Einsele, Stefan O. Ciurea, Ciprian Tomuleasa

**Affiliations:** 1 Department of Personalized Medicine and Rare Diseases, Medfuture Institute for Biomedical Research, Iuliu Hațieganu University of Medicine and Pharmacy, Cluj-Napoca, Romania; 2 Department of Hematology, Iuliu Hațieganu University of Medicine and Pharmacy, Cluj-Napoca, Romania; 3 Department of Hematology, Ion Chiricuta Oncology Institute, Cluj-Napoca, Romania; 4 Department of Oncology, Bistrita Emergency Hospital, Bistrita, Romania; 5 Department of Nanosciences, Medfuture Institute for Biomedical Research, Iuliu Hațieganu University of Medicine and Pharmacy, Cluj-Napoca, Romania; 6 Faculty of Physics, Babeș-Bolyai University, Cluj-Napoca, Romania; 7 Department of Leukemia, Sidney Kimmel Comprehensive Cancer Center, Johns Hopkins University School of Medicine, Baltimore, MD USA; 8 Department of Stem Cell Transplantation, Fundeni Clinical Institute, Bucharest, Romania; 9 Department of Hematology, Carol Davila University of Medicine and Pharmacy, Bucharest, Romania; 10 Department of Internal Medicine II, Julius Maximilians University of Würzburg, Würzburg, Germany; 11 Hematopoietic Stem Cell Transplantation and Cellular Therapy Program, Division of Hematology/Oncology, Department of Medicine, University of California Irvine, Orange, CA USA

**Keywords:** Minimal residual disease, Acute myeloid leukemia, SERS, DNA methylation profiling

## Abstract

Measurable residual disease (MRD) assessment has become a cornerstone in the management of acute myeloid leukemia (AML), offering critical prognostic information and guiding post-remission therapy. Conventional MRD detection methods, including multiparameter flow cytometry (MFC), quantitative PCR (qPCR), and next-generation sequencing (NGS), have demonstrated strong predictive value but are limited by technical complexity, marker specificity, and accessibility. This review explores the current landscape of MRD monitoring in AML, covering cytogenetic, immunophenotypic, and molecular approaches, with particular emphasis on the strengths and limitations of each. We further examine promising emerging technologies—namely DNA methylation profiling and surface-enhanced Raman scattering (SERS)—as non-invasive alternatives. DNA methylation-based assays capitalize on the epigenetic dysregulation characteristic of AML, while proof-of-concept studies indicate SERS as a promising alternative for cancer subtypes, stages or specific mutation detection by analyzing biofluids or extracted DNA from blood. Together, these developments hold the potential to overcome current diagnostic limitations, enabling more universal and precise MRD assessment. Ongoing research and validation will determine their future integration into standard clinical practice.

## MRD in AML

Despite significant progress over recent decades—including the approval of more than a dozen chemotherapies and the established use of allogeneic hematopoietic stem cell transplantation (HSCT) —acute myeloid leukemia (AML) remains a challenging hematologic malignancy [1]. These therapies often lead to complete remission (CR), which is defined as having less than 5% blasts in the bone marrow (BM), normal peripheral blood counts, and no signs of disease outside the BM [2, 3]. However, 30–50% of patients who achieve CR eventually relapse. This suggests that small numbers of leukemic stem cells (LSC) persist after treatment, which cannot be detected by standard morphological methods. To address this limitation, the concept of MRD has emerged, referring to the detection of leukemic cells at levels below the threshold of conventional morphological assessment [3–5]. Approved methods for assessing MRD status include MFC and molecular techniques such as conventional qPCR, digital PCR (dPCR), and NGS. These methods are approved by the European LeukemiaNet, the EuroMRD Consortium and the EuroFlow consortium [3–6].

The 2021 ELN guidelines for MRD assessment recommend the use of quantitative qPCR in patients with established molecular MRD markers, while MFC is advised for all other cases [6]. Currently, validated molecular MRD markers are primarily available for patients classified as favorable risk according to the 2022 ELN risk stratification, including NPM1 mutations, t(8;21)/RUNX1::RUNX1T1, inv [16]/CBFB::MYH11, and t(15;17)/PML::RARα [6].

Beyond the recommended methods, additional techniques—including cytogenetics, DNA methylation analysis, and SERS-based technologies—have also been explored and will be discussed in this review. These emerging approaches hold the potential to revolutionize MRD monitoring in the future. Therefore, our state-of-the-art review analyzes and compares various MRD detection methods, with a particular focus on evaluating the clinical potential and validation requirements of DNA methylation–based assays and SERS.

## Cytogenetics for MRD

**Fig. 1:**
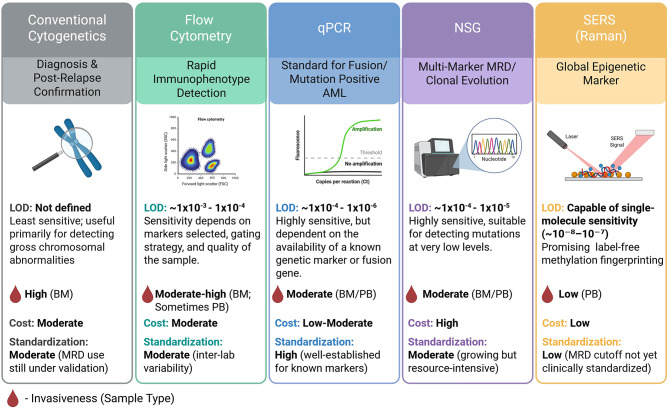
Comparison of currently available MRD-assessment tools. LOD – limit of detection, BM – bone marrow, PB – peripheral blood

Cytogenetic assessment of MRD in AML, conceptually an extension of morphological evaluation, relies on detecting abnormal karyotypes in patient blood or bone marrow using techniques such as G-banding or fluorescence in situ hybridization (FISH) [7]. Most chromosomal abnormalities identified in AML fall into two main categories, based on whether there is a net gain or loss of genetic material: balanced structural abnormalities—such as translocations and inversions—which do not result in a net gain or loss, and unbalanced structural aberrations—such as deletions, duplications, and monosomies—which lead to a loss or gain of genetic material. The most common examples of abnormalities are shown in Table 1; Fig. 1. Moreover, the limits of detection (LoD) for cytogenetics is not well defined, while the LoD for MFC is 10^− 3^-10^− 4^, for qPCR up to 10^− 6^, NGS up to 10^− 5^ with high sensitivity in detecting mutations at very low levels, and in the case of SERS the LoD is up to 1 × 10^− 6^ as per experimental studies, offering a promising label-free methylation fingerprinting. All the details are depicted in Fig. 1 [8–12] (Table 2).

**Table 1:** Chromosomal abnormalities in acute myeloid leukemia (AML) [13]

Category	Abnormalities	Frequency / Notes
Common Balanced Abnormalities	• t(8;21)(q22;q22) / AML1::ETO • t(15;17)(q22;q12) / PML::RARα • inv(16)(p13q22) or t(16;16)(p13;q22) / CBFβ::MYH11 • 11q23 rearrangements	Found in 15–25% of AML patients
Less Common Balanced Abnormalities	• inv(3)(q21q26), t(3;3)(q21;q26) • t(6;9)(p23;q34) / DEK::CAN • t(8;16)(p11;p13) / MOZ::CBP • t(1;3)(p36;q21) • t(1;22)(p13;q13) • t(3;21)(q26;q22) • t(7;11)(p15;p15) • t(11;17)(q23;q25) • t(16;21)(p11;q22)	Rare, but relevant in specific AML subtypes
Unbalanced Structural Abnormalities	• Monosomy: -7 • Trisomy: +8, + 11, +13, + 21 • Deletions: 5q, 7q, 9q	Represent net gain or loss of genetic material
Complex Karyotypes	Defined as three or more chromosomal abnormalities	Present in 10–20% of AML cases; associated with poor prognosis

**Table 2:** Comparison of currently available MRD assessment tools

Tool	Sensitivity	Specificity	Sample	Validation	Time	Cost	Limitations
MFC [67]	10^− 3^-10^− 4^	90–98%	> 500 µl	multicentric	hours	moderate	standardization
qPCR/ dPCR [68, 69]	10^− 4^-10^− 6^	98–100%	> 500 µl	multicentric	hours	moderate	-needs validated molecular marker -requires assay design.
NGS [70]	10^− 4^-10^− 6^	95–99%	> 300 µl	multicentric	days	high	-Expensive analysis expertise;
Cyto-genetics [71]	10^− 1^-10^− 2^	100%	1–2 ml	multicentric	days	low	-Lower sensitivity -contradictory findings across studies. -LOD is often not defined.
DNA methy-lation-based assays [72, 73]	10^− 3^-10^− 4^	90–98%	< 300 µl	Pilot studies only	hours	moderate	- some methylation patterns overlap with normal hematopoietic cells - presence of CHIP - lack of standardization
SERS [9, 74]	10^− 6^-10^− 5^	90–99%	< 100 µl	No validation yet	Within 1 min	low	Only proof-of-concept studies; no standard operating procedure;

The efficacy of cytogenetic methods for MRD assessment was tested in the context of HSCT. A study assessed MRD status in a cohort of AML patients before HSCT. The authors demonstrated that conventional cytogenetic methods like G-banding and FISH were just as effective as MFC in assessing MRD and predicting outcomes and survival of patients. The results of the study showed that MRD positive patients (with both MFC and cytogenetics) had significantly higher relapse rates, lower progression-free (PFS) and overall survival (OS), thereby consolidating the role of cytogenetics in assessing MRD status in AML. However, the role of cytogenetics for MRD status in AML still needs further characterization and validation, since other studies are reporting contradictory findings [14].

## Multiparametric flow cytometry for MRD

Immunophenotyping by MFC is the most utilized technique for MRD assessment in AML and other blood cancers. MFC-MRD consists of two distinct approaches: the Leukemia-Associated Immunophenotype (LAIP) approach and the Different from Normal approach (DfN) [1, 15].

The LAIP approach involves the continuous monitoring of a specific immunophenotypic profile established at the time of diagnosis. A key limitation of the LAIP approach is its reliance on a pre-chemotherapy BM sample to define the initial leukemic immunophenotype. Furthermore, the initial phenotype may change over time, leading to the emergence of leukemic subclones that are not captured by the original LAIP, thereby reducing the method’s reliability for long-term monitoring [4].

In contrast, the DfN approach consists of determining a leukemic phenotype in comparison with normal cells. The leukemic phenotype might be represented by the over or under-expression of certain antigens and the presence of aberrant markers [4]. The DfN approach is therefore able to identify abnormal cells regardless of changes in the initial leukemic phenotype by identifying certain niches in the BM and PB that are not normally occupied by cells under physiological conditions.

For MRD detection in AML using MFC, current guidelines recommend a harmonized approach that integrates both LAIP and DfN strategies [6]. This integrated method relies on a core panel of markers—CD34, CD117, CD45, CD33, CD13, CD56, CD7, and HLA-DR—for the standardized assessment of all samples. The inclusion of CD38 is also supported, as it enhances specificity in distinguishing aberrant immunophenotypes, particularly within the CD34⁺CD38^low^ compartment. In such cases, co-expression of additional markers like CD56, CD7, or CD45RA—typically associated with leukemic stem cells—may further support MRD identification. In AML subtypes with a monocytic component, supplementary markers such as CD64, CD11b, and CD4 are recommended to improve detection accuracy and phenotypic resolution. Detection of novel disease biomarkers, such as C-type lectin-like molecule-1 (CLL-1) and galectin-9 or the use of nanosensors for circulating DNA, microRNAs and leukemia-specific proteins might also significantly improve sensitivity of MFC-assays [16–19].

One of the prominent advantages of MFC is represented by its accessibility. Indeed, the method was determined to be applicable for over 90% of AML cases, providing results within a relatively short period of time (up to 2 weeks), with an acceptable sensitivity of 10^− 3^ to 10^− 4^. Another advantage of MFC is represented by the ability to discern viable cells from dead cells or other debris. The limitations of this method are represented by the need for fresh and high quality samples for assessment, poor reproducibility and standardization between different laboratories, and the need for substantial expertise to accurately interpret the results [20].

A large multicenter pediatric AML study involving 524 patients revealed that MFC measured at two timepoints from BM samples — first between days 28–35 post-induction (TP1) and end of second induction (TP2) — was prognostic when using a 0.1% threshold. Notably, while TP1-MRD positivity correlated with inferior PFS and OS, TP2-MRD emerged as the stronger independent predictor across intermediate- and high-risk groups (including KMT2A rearrangements), was independent in multivariate models, and guided decisions about early HSCT vs. consolidation chemotherapy [21]. In contrast, it was reported that “indeterminate” MRD results—typically low-level aberrant immunophenotypes (~ 0.01–0.1%) overlapping with a regenerating BM—constituted about 35% of assays. This group demonstrated significantly higher relapse rates (~ 21%) than MRD-negative patients (8%), but lower than clearly MRD-positive ones (33%), suggesting that indeterminate MRD signals categorize an intermediate-risk group meriting closer monitoring [22]. Together, these studies illustrate the nuanced value of MFC: definitive positivity clearly stratifies high-risk, while indeterminate results in adults may carry moderate relapse-risk—emphasizing the importance of standardized thresholds and integrated clinical algorithms post-induction.

The role of MFC-MRD assessment was also assessed in the context of allogenic HSCT in AML. The results of the study proved that pre-HSCT MRD status on BM samples has a significant impact on relapse rates and OS of patients, MRD-positivity being associated with higher risk of relapse and shorter survival. Moreover, the authors found that the “low MRD” (0.1–0.5%) subgroup had no significantly different effects on relapse risk in comparison with “high MRD” (>0.5%) [23].

## Molecular MRD assessment in AML patients

The molecular MRD approach encompasses two assays: PCR and NGS. One major advantage of PCR-based MRD assessment compared to MFC is its superior sensitivity, with a lower limit of detection ranging from 10⁻⁴ to 10⁻⁶. However, despite this high sensitivity, the method presents certain limitations—most notably, its applicability is restricted to patients harboring specific gene fusions and/or mutations. Given the genetic heterogeneity of AML, PCR-based MRD detection is feasible in only about 50% of cases [1].

Thus, the main molecular targets monitored by qPCR are represented by gene fusions such as RUNX1::RUNX1T1, CBFB::MYH11 and PML::RARα. KMT2A::MLLT3 and DEK::NUP214 are other gene fusions specific to AML, though their use as molecular markers is quite rare since qPCR protocols are less validated [1, 6].

In addition to gene fusions, several other molecular markers are amenable to MRD detection by PCR. Among these, *NPM1* mutations are the most prevalent, occurring in approximately 25–35% of AML cases. These mutations most commonly involve a 4-base pair insertion in exon 12, which provides a stable and leukemia-specific target for MRD monitoring. *WT1* overexpression is another frequently used marker; however, its application is limited by the lack of a standardized threshold and high physiological background expression, which can compromise specificity [6]. Mutations in *IDH1* and *IDH2*, detected in around 20% of AML patients, and *FLT3-ITD* mutations are also of clinical interest—not only for MRD assessment but also as therapeutic targets. Nonetheless, *FLT3* mutations are known to be unstable between diagnosis and relapse, which may hinder their reliability for longitudinal MRD monitoring. Despite these limitations, the inclusion of these molecular markers remains valuable, particularly when used in conjunction with other modalities.

NGS allows for a quick and easy assessment of genomic features, and it is a powerful tool for diagnosing AML subtypes and identifying MRD. The main advantage of the method is that it does not require *a priori knowledge* of the mutation status. Current guidelines recommend a core panel of approximately two dozen genes that collectively cover a large proportion of AML cases and may be effectively used to support a panel-based strategy for MRD monitoring [6]. The most important recommendations are the following: First, germline mutations must be carefully excluded to avoid confounding the interpretation of MRD results. Moreover, while DTA mutations (mutations in *DNMT3A*,* TET2* and *ASXL1* genes) can be present in leukemic cells, they can also be found in age related clonal hematopoiesis and their exclusion from the analysis is recommended. In addition to *DNMT3A*,* TET2* and *ASXL1*, in CHIP and MDS other genes can acquire mutations. *SF3B1* and *SRSF2* mutations are associated with MDS, in elderly patients, while *JAK2* mutations are representative for CHIP and myeloproliferative syndromes. *TP53*,* PPM1D* and *IDH1/2* are mutated in CHIP and MDS and are associated with clonal expansion and risk of proliferation towards hematological malignancies, but not exclusive to AML [24]. Clonal dynamics evaluation using whole-exome and targeted sequencing of 699 patients compared secondary acute myeloid leukemia (sAML) to high-risk MDS and highlighted several newly acquired mutations in *FLT3*,* PTPN11*,* WT1*,* IDH1*,* IDH2* and *NRAS*. On the other hand, comparing high-risk MDS with low-risk MDS revealed mutated genes with weaker impact on sAML progression and overall survival (OS) including *TP53*,* GATA2*,* KRAS*,* RUNX1*,* STAG2*,* ASXL1*,* ZRSR2* and TET2 [25]. However, if detectable mutations are only present in DTA genes, MRD should be assessed using other methods such as MFC or qPCR. Furthermore, mutations in genes involved in signaling pathways like *FLT3*, *KIT*,* KRAS*,* NRAS* might be sub-clonal and have a low negative predictive value, according to 2021 and 2024 ELN updates for MDS and AML, even though they could indicate the presence of residual AML cells. Therefore, these mutations should not be used as NGS-MRD markers alone, but in combination with other MRD markers. Additionally, patients undergoing AML therapy with targeted agents such as FLT3 or IDH1/IDH2 inhibitors should have the specific targeted gene as an NGS-MRD marker, in combination with other markers that are present in the sample [6].

Practical implementation of NGS-MRD requires careful mutation selection, guided by the biological role of each variant and its stability during disease evolution. According to the 2021 ELN MRD consensus and 2024 ELN risk update, only mutations clearly associated with leukemic clones—such as NPM1, FLT3-ITD/TKD, IDH1/2, KIT, RUNX1, WT1, NRAS/KRAS, and TP53—should be routinely used for MRD tracking. In contrast, mutations typical of age-related clonal hematopoiesis (DNMT3A, TET2, ASXL1) are excluded from MRD interpretation because their persistence after therapy does not necessarily imply residual leukemia [6, 26].

When persistent DTA variants co-exist with a diagnostic driver mutation, the latter should be used for quantitative follow-up, while DTA mutations may inform clonal dynamics or predispositions to relapse but not relapse per se. For newly diagnosed AML, comprehensive panel testing (20–40 genes) is recommended to identify candidate MRD targets. Once CR is achieved, patient-specific minimal panels focusing on stable driver mutations can be applied longitudinally to reduce cost and turnaround time. Persistent or re-emergent non-DTA mutations above 0.1–1% variant allele frequency (VAF) are clinically meaningful and warrant closer monitoring or pre-emptive therapy. Conversely, isolated DTA persistence at low VAF may represent clonal hematopoiesis of indeterminate potential (CHIP) and should not, by itself, trigger therapeutic intervention.

The efficiency of NGS in assessing clinical decisions and MRD status in AML was proven by a series of studies. First, a study involving a cohort of 162 AML patients demonstrated that proper NGS gene panels are efficient in identifying and diagnosing different AML subtypes, being able to guide therapeutic decisions. The NGS panel performed on BM samples was able to identify 339 relevant mutation variants in 18 out of the 19 targeted genes. Most of the mutations were SNVs and the most frequently mutated gene was *NPM1*. The sensitivity of the NGS panel was assessed by comparison with data obtained from conventional molecular biology techniques from the same patients. The authors found that the NGS panel had a sensitivity and specificity of 100%, being able to identify all the mutations detected by conventional molecular biology techniques. Moreover, the NGS panel was able to detect 17 additional mutations that were not identified conventionally [27].

Boudry et al., measured the MRD in AML on 98 adults in first CR after intensive chemotherapy using duplex UMI-based NGS capture panel. The NGS-MRD combined with MFC-MRD identified a double-positive subgroup with poor outcomes, highlighting the sensitivity of the NGS methods [28]. Moreover, the study coordinated by Patkar et al., evaluated the MRD in 201 patients treated with conventional therapy, in CR. The NGS-MRD identified over 80% of the cases identified by MFC at end of induction while FCM identified 49.3% of the MRD identified by NGS, with only a small number of NGS-MRD negative but FCM-MRD positive samples. Their study highlighted the need for a combined evaluation using both FCM and NGS-MRD for a maximum clinical utility [29].

Furthermore, the efficacy of NGS assessment of MRD in AML was also determined in the context of allogenic HSCT. The authors of the study monitored the MRD status using NGS in a cohort of AML patients in morphologic CR before allogenic HSCT. They discovered that the technique has a wide applicability for AML patients and a high predictive value of disease relapse and survival, having a definite role in the post-HSCT management of patients [30]. Some of the significant advantages of NGS for MRD assessment are represented by its high sensitivity of 10^− 4^-10^− 5^, and its customizability, making it a more personalized approach [31]. Forty-two AML patients were evaluated for the utility of MRD detection using NGS-based method, in a study conducted by Press et al. The patients underwent serial disease monitoring both by standard methods and NGS assay for 42 genes, to detect mutations specific to AML in BM samples. Their results showed that NGS assay has a high sensitivity compared to non-molecular methods, with 28 MRD-positive subjects compared to 11 MRD-positive subjects. Thirteen of them relapsed after HSCT and only one was MRD negative, fact that underscores the high sensitivity of NGS-MRD and its utility in the case of HSCT [32]. Others have questioned if DNA sequencing from blood samples collected from AML patients in first CR prior to HSCT could identify the patients at risk for subsequent relapse. The study evaluated 371 patients in first CR who received HSCT and showed that the presence of FLT3 internal tandem duplication (FLT3/ITD) and *NPM1* variants before HSCT were associated with increase rates of relapse and poor survival, results that also underscore the need for NGS-MRD assessment to obtain best outcomes in AML patients [33]. However, the use of NGS for MRD assessment is still limited by its high operating cost and expertise for data analysis [6].

## Emerging techniques for MRD monitoring – DNA methylation and SERS

### DNA methylation and MRD monitoring in AML

The genomic and phenotypic diversity of AML has driven the search for alternative strategies that can address the limitations of conventional MRD assessment methods. Among these, DNA methylation has emerged as one of the most promising approaches.

In AML, DNA methylation is markedly altered, both globally and at specific genomic loci [34–36]. These disruptions offer valuable potential for improving the sensitivity and specificity of MRD detection.

**Fig. 2:**
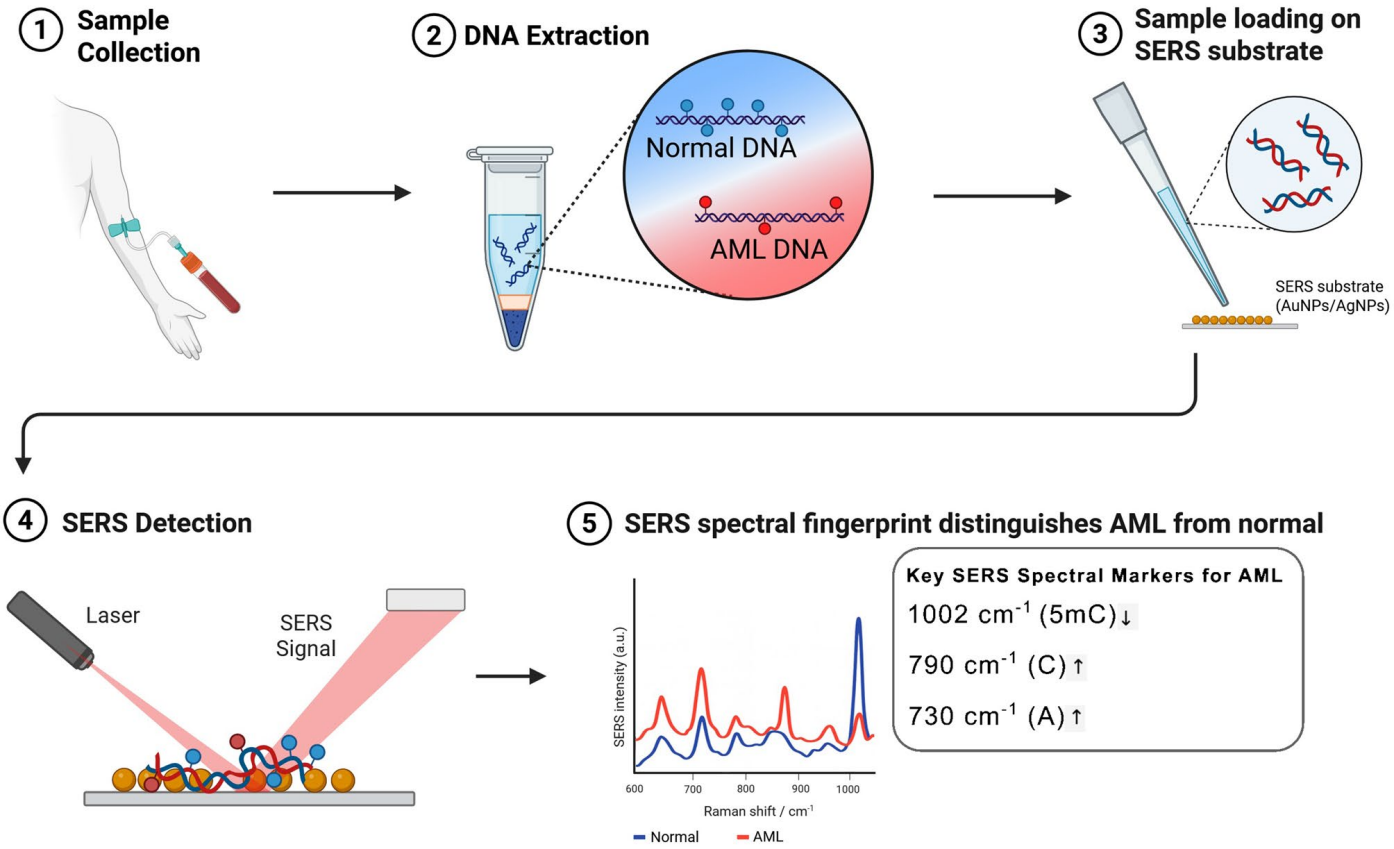
Workflow for SERS analysis of DNA methylation patterns. The schematic illustrates the stepwise process for detecting DNA methylation using SERS: [1, 2] Blood sample collection and DNA extraction [3]. The extracted DNA is loaded onto a metallic SERS substrate (e.g., AuNPs/AgNPs) [4]. Laser excitation of the DNA-metal mixture to acquire the SERS spectrum of DNA [5] The resulting SERS spectral fingerprint distinguishes AML DNA from normal control DNA based on characteristic spectral differences

The critical role of DNA methylation in AML is underscored by the high frequency of mutations in genes encoding epigenetic regulators, particularly those involved in DNA methylation and demethylation pathways [35]. DNA methylation is a covalent biochemical process in which methyl groups are transferred to the 5-carbon position of cytosine residues, predominantly within CpG dinucleotides, resulting in the formation of 5-methylcytosine (5-mC) [37]. This reaction is catalyzed by DNA methyltransferases (DNMTs), using S-adenosyl-L-methionine (SAM) as the methyl group donor. DNMTs are categorized into two functional classes: de novo methyltransferases (DNMT3A and DNMT3B), which establish new methylation marks, and maintenance methyltransferases (mainly DNMT1), which preserve existing methylation patterns during DNA replication [38]. CpG islands—often located in gene promoter regions—are typically unmethylated under physiological conditions, allowing active gene transcription [39]. Methylation at these sites represses gene expression through multiple mechanisms: it can reduce the binding affinity of transcription factors and promote a closed chromatin conformation via recruitment of histone deacetylases and methyltransferases.

Dysregulation of this finely tuned system is a hallmark of cancer. AML cells frequently exhibit aberrant DNA methylation, characterized by global hypomethylation and focal hypermethylation, particularly in tumor suppressor gene promoters [40–42]. Among the most commonly mutated epigenetic regulators in AML is DNMT3A, altered in approximately 15–35% of patients, leading to disrupted methylation landscapes [1, 35]. Counterbalancing DNA methylation, the ten-eleven translocation (TET) family of enzymes—particularly TET2—mediate active DNA demethylation by sequentially oxidizing 5-mC to 5-hydroxymethylcytosine (5-hmC), 5-formylcytosine (5-fC), and 5-carboxycytosine (5-caC) [43]. TET enzymes require 2-oxoglutarate as a cofactor and are inhibited by succinate. Reduced expression or loss-of-function mutations in TET2 have been identified in various hematologic malignancies, including AML, and are associated with decreased levels of 5-hmC and altered epigenetic regulation [43]. Experimental studies using Dnmt3a^–/–^ Tet2^–/–^ double knockout mouse models have demonstrated accelerated leukemogenesis, further confirming the cooperative role of these mutations in AML pathogenesis [44, 45]. Moreover, TET2 function is intricately linked to the activity of *IDH* genes, which are frequently mutated in AML [46]. In its wild-type form, IDH produces α-ketoglutarate, a cofactor that stimulates TET activity [47]. However, mutant IDH enzymes generate 2-hydroxyglutarate, a structural mimic that competitively inhibits TET function [48], thereby contributing to epigenetic dysregulation. This mechanistic insight has led to the development of targeted therapies such as IDH inhibitors. For the moment, IDH inhibitors are approved in AML both frontline (for patients ineligible for intensive regimens) and in the relapsed/refractory setting [26, 49–53].

In line with the paramount importance of methylation changes in AML transformation, several studies demonstrated the possibility to implement MRD testing by methylation markers. One such study investigated aberrant methylation at neighboring CpG sites to discriminate between healthy and malignant samples in AML and myelodysplastic syndromes (MDS). Using targeted pyrosequencing of the *PDE4C* gene region, the authors developed two scoring metrics—the delta score (d-score) and neighborhood score (n-score)—to quantify methylation variability. These scores were significantly elevated in samples from patients at AML diagnosis and relapse, and normalized during CR, indicating that they can reflect disease burden. Furthermore, these scores correlated with overall survival in MDS patients, suggesting prognostic utility [54].

Building on this, a subsequent study evaluated the role of DNA methylation patterns in assessing MRD in AML. By identifying four CpG sites frequently aberrant in AML and applying bisulfite amplicon sequencing (BA-seq), the authors developed an “AML-score” to distinguish malignant from healthy samples. Despite high accuracy in initial diagnosis, the score and machine learning-based classifiers (random forest and autoencoders) had limited ability to reliably distinguish MRD-positive from MRD-negative samples, primarily due to background methylation variability in healthy controls. Nonetheless, the approach demonstrated promise for MRD estimation in patients lacking molecular markers and highlighted the need for improved sensitivity or integration with existing methodologies [55].

### SERS for MRD

The hematologic nature of AML has sparked interest in analytical techniques that evaluate the molecular composition of blood. One such approach is surface-enhanced Raman scattering (SERS), an enhanced method of the Raman effect [56]. SERS detects the specific vibrational modes of molecules, producing a unique spectral fingerprint that provides detailed insights into chemical composition, molecular structure, and intermolecular interactions. Its key advantage over conventional Raman spectroscopy lies in its significantly enhanced sensitivity, achieved through the use of metal nanoparticles—typically silver or gold—that amplify the Raman signal of target molecules by several orders of magnitude [20, 57].

MRD detection through direct detection of cells using SERS is limited with LOD down to 10^− 6^ M [9], as SERS sensibility decreases with distance to the metallic structure and therefore is not able to analyze the cell whole composition. More commonly, SERS investigates MRD in an indirect manner, through DNA alterations or metabolic profile of biofluids [8, 9], which could improve the LOD of MRD using SERS. In this approach, it is difficult to directly compare SERS with other techniques in terms of its detection limit, since no direct cell counting is performed and only the indirect effects of minimal residual disease are analyzed.

Most research in this field focuses on diagnosis and subtyping — including AML — using biofluid samples. These offer the advantage of minimal preprocessing steps and enable rapid analysis within one minute. A notable example is the retrospective study by Xiong et al.., which demonstrated the effectiveness of a SERS-based approach using silver nanoparticles for the detection of various cancers, including AML (20 AML samples and 30 healthy controls), directly from patient serum samples [58]. Combining SERS with deep learning algorithms AML cases were identified with 100% accuracy.

Complementing these findings, Oktem et al.. further validated the utility of SERS in AML by analyzing blood-derived samples using a similar approach [59]. Their retrospective study confirmed that SERS can reliably distinguish AML patients from healthy controls (43 AML samples and 24 healthy controls), with an accuracy of 95.4% reinforcing its diagnostic value. In addition, the study highlights the potential of SERS in differentiation between AML and ALL by analyzing blood from 24 healthy donors, 43 AML patients and 18 ALL. More importantly, the study extended the potential application of SERS blood analysis to risk stratification and monitoring of disease recurrence. By identifying spectral features associated with relapse-prone patients, the authors suggested that SERS could serve as a minimally invasive tool for MRD assessment or early relapse prediction—two major clinical challenges in AML management. This idea was further investigated in small cohort of 49 APL patients [60]. These studies illustrate how the integration of nanotechnology and artificial intelligence can advance non-invasive, label-free diagnostic methods with clinical applicability.

Given that global changes in DNA methylation lead to widespread molecular alterations in AML, the genomic DNA of transformed cells exhibits distinct molecular signatures that can be detected using SERS (Fig. 2). In a retrospective study by Moisoiu et al.., which included 17 DNA samples extracted from the peripheral blood of AML patients and 17 from controls, the authors demonstrated that cytosine and 5-methylcytosine (5-mC) produce distinguishable SERS spectra [61]. Notably, 5-mC exhibited a characteristic spectral band at 1002 cm⁻¹, corresponding to the vibrational mode of its methyl group. This spectral feature was prominent in the genomic DNA of healthy individuals but appeared significantly reduced in DNA from AML patients, consistent with the global hypomethylation typically observed in AML cells. The study successfully detected these spectral differences between AML and normal genomic DNA, highlighting the potential of SERS as a non-invasive, label-free method for AML diagnosis and monitoring based on epigenetic signatures. Besides direct analysis of cytosine methylation SERS bands, the differences in DNA methylation patterns associated with AML are also revealed by adenine SERS bands. Because SERS is sensitive to the interaction of DNA with the metallic substrate, changes in methylation pattern can influence the physicochemical absorption of DNA and result in shifts or intensity variations in the SERS bands of other DNA bases [60–62]. However, the SERS analysis of DNA methylation does not reflect exact position of the 5-mC.

Although single mutations have been reported to be detectable by SERS [63], NGS remains the gold standard for MRD assessment until the above-mentioned limitations are addressed, as it enables mutation tracking with very high specificity and precision [64].

Given the complexity of DNA methylation and its variability across physiological and pathological conditions, the integration of SERS with already established diagnostic methods may be required to ensure reliability and clinical utility [55, 65, 66]. A notable study employed a SERS-based approach that combined hollow-core photonic crystal fibers (HC-PCFs) with silver nanoparticles to enhance signal sensitivity and reproducibility [8]. This system enabled highly sensitive detection of AML cells, demonstrating strong signal amplification and the ability to differentiate malignant from non-malignant cells based on their unique Raman spectra. What makes this approach particularly compelling is that it positions SERS not only as an alternative to molecular MRD assessment, but also as a potential substitute for MFC. Unlike MFC, which requires viable cells and antibody panels targeting known surface markers, the SERS method is label-free and relies on intrinsic molecular signatures—offering a broader, potentially more unbiased detection strategy. This is especially relevant in cases where AML cells downregulate typical surface markers or when sample viability is compromised.

Together, these proof-of-concept studies underscore the promise of SERS as a diagnostic platform for AML. Unlike traditional MRD methods that rely on MFC, PCR, or sequencing, SERS offers a non-invasive, rapid, and cost-effective alternative that can be applied directly to biofluids. The ability to pair this technique with advanced computational models adds another layer of precision, potentially enabling real-time clinical decision-making in diagnosis, prognosis, and treatment monitoring. Although SERS shows great promise for the analysis of biofluids and DNA in AML detection as well as for MRD monitoring, several limitations remain. The available studies are retrospective and have been conducted in small, unicentric cohorts. In addition, none of the studies reported external validation of the results. However, in the absence of a standard operating procedure the improvement of the technique toward clinical applications will be limited. Further validation in larger, multi-center cohorts and external validation in prospective studies will be critical in translating these findings into clinical practice.

## Conclusions

Despite advances in chemotherapeutic regimens, targeted therapies, and HSCT, relapses remain a significant challenge in AML, underscoring the critical need for effective and accessible MRD monitoring. Current methodologies—MFC, PCR, and NGS—have improved risk stratification and treatment personalization but are constrained by their reliance on specific markers, technical expertise, and biological sample quality. As such, alternative approaches are increasingly explored. Cytogenetic analysis, though traditionally considered limited in sensitivity, has demonstrated prognostic utility in certain settings and may complement other MRD techniques. Emerging technologies, particularly DNA methylation profiling and SERS, represent exciting avenues for broader and more sensitive MRD detection. DNA methylation-based assays exploit the epigenetic disruptions intrinsic to AML pathogenesis, offering novel biomarker potential even in genetically heterogeneous cases. Meanwhile, SERS might be a non-invasive platform capable of detecting disease-specific molecular signatures with high sensitivity—potentially serving as an alternative to both molecular and MFC-based MRD assessments, but it’s applications are at the very beginning of clinical validation or invalidation and thus thorough studies on large patient cohorts are mandatory. As these approaches advance and integrate with machine learning and nanotechnology, they offer the prospect of more comprehensive, real-time MRD evaluation. Ultimately, the future of MRD monitoring in AML lies in a multimodal, personalized approaches that combine complementary technologies such as MFC, NGS, and epigenetic profiling. While each of these modalities provides unique insights into residual disease biology— MFC capturing phenotypic aberrancies, NGS revealing clonal evolution at the genomic level, and epigenetic assays detecting stable, lineage-specific methylation signatures—their combined application could markedly enhance sensitivity, specificity, and biological interpretability. Such integrative strategies may enable more accurate disease monitoring across heterogeneous patient populations and treatment regimens. To translate these advances into clinical practice, prospective clinical trials are essential to demonstrate the prognostic and therapeutic value of MRD-guided interventions, including risk-adapted treatment intensification or de-escalation. These studies should also evaluate the timing, thresholds, and clinical decision algorithms linked to MRD positivity across different assay platforms. Finally, the establishment of clear regulatory pathways and standardized validation frameworks will be crucial to support the diagnostic adoption of multimodal MRD testing. Collaboration between academic centers, industry, and regulatory agencies will be required to define performance benchmarks, ensure analytical reproducibility, and facilitate clinical implementation, ultimately enabling MRD to serve as a robust biomarker for precision medicine in hematologic malignancies.

## Data Availability

All data and material are available upon request.

## Abbreviations

MRD: Measurable residual disease
AML: Acute myeloid leukemia
MFC: Multiparameter flow cytometry (MFC)
qPCR: Quantitative PCR
NGS: Next-generation sequencing
SERS: Surface-enhanced Raman scattering
HSCT: Hematopoietic stem cell transplantation
CR: Complete remission
BM: Bone marrow
LSC: Leukemic stem cells
dPCR: Digital PCR
FISH: Fluorescence in situ hybridization
LOD: Limit of detection
PB: Peripheral blood
LAIP: Leukemia-Associated Immunophenotype
DfN: Different from Normal approach
OS: Overall survival
SAM: S-adenosyl-L-methionine
TET: Ten-eleven translocation
5-hmC: 5-hydroxymethylcytosine
5-fC: 5-formylcytosine
5-caC: 5-carboxycytosine
